# Cross-Representational Signaling and Cohesion Support Inferential Comprehension of Text–Picture Documents

**DOI:** 10.3389/fpsyg.2020.592509

**Published:** 2021-01-18

**Authors:** Juliette C. Désiron, Mireille Bétrancourt, Erica de Vries

**Affiliations:** ^1^Technologies de Formation et Apprentissage (TECFA), Faculty of Psychology and Education, University of Geneva, Geneva, Switzerland; ^2^LaRAC, Univ. Grenoble Alpes, Grenoble, France

**Keywords:** multimedia learning, text cohesion, signaling, comprehension, inference, eye-tracking

## Abstract

Learning from a text–picture multimedia document is particularly effective if learners can link information within the text and across the verbal and the pictorial representations. The ability to create a mental model successfully and include those implicit links is related to the ability to generate inferences. Text processing research has found that text cohesion facilitates the generation of inferences, and thus text comprehension for learners with poor prior knowledge or reading abilities, but is detrimental for learners with good prior knowledge or reading abilities. Moreover, multimedia research has found a positive effect from adding visual representations to text information, particularly when implementing signaling, which consists of verbal or visual cues designed to guide attention to the pictorial representation of relevant information. We expected that, as with text-only documents, struggling readers would benefit from high text cohesion (Hypothesis 1) and that signaling would foster inference generation as well (Hypothesis 2). Further, we hypothesized that better learning outcomes would be observed when text cohesion was low and signaling was present (Hypothesis 3). Our first experimental study investigated the effect of those two factors (cohesion and signaling) on three levels of comprehension (text based, local inferences, global inferences). Participants were adolescents in prevocational schools (*n* = 95), where some of the students are struggling readers. The results showed a trend in favor of high cohesion, but with no significant effect, a significant positive effect of cross-representational signaling (CRS) on comprehension from local inferences, and no interaction effect. A second experiment focused on signaling only and attention toward the picture, with collection of eye-tracking data in addition to measures of offline comprehension. As this study was conducted with university students (*n* = 47), who are expected to have higher reading abilities and thus are less likely to benefit from high cohesion, the material was presented in its low cohesive version. The results showed no effect of conditions on comprehension performances but confirmed differences in processing behaviors. Participants allocated more attention to the pictorial representation in the CRS condition than in the no signaling condition.

## Introduction

The use of text–picture combinations has become increasingly common in instructional documents in school and everyday life. However, learners with low reading abilities struggle to comprehend instructional texts (e.g., Cain and Oakhill, 2014). Text processing research has defined struggling readers as learners who have trouble both decoding and comprehending a text (Hoover and Gough, 1990; Kenedou et al., 2010; Florit and Cain, 2011). A previous study by Désiron (unpublished) showed that for young adults, language comprehension abilities (vocabulary and verbal reasoning) were predictors of multimedia comprehension, but decoding abilities were not. These results were in line with text processing research that confirmed Kintsch’s construction–integration theory (Kintsch and van Dijk, 1978; Kintsch, 1980) in that manipulating text cohesion to support the generation of inferences positively affected comprehension of a text-only document (e.g., McNamara et al., 1996; Ozuru et al., 2009). In a text–picture, or multimedia, document, the need to generate inferences also occurs between the text and the picture (Holmes, 1987) and has been theorized as the need to create links between the text and the picture to integrate them together and form a coherent mental model (Mayer, 2014; Schnotz, 2014). Similar to text cohesion, the signaling principle of multimedia learning considers that text–picture integration and thus comprehension can be facilitated by visually elucidating the link between both representations (van Gog, 2014). Numerous studies have showed the positive effect of signaling on comprehension, particularly for learners with little prior knowledge (see Richter et al., 2017 for a review). Hence, changes in the cohesion of the text and the use of signaling between the text and the picture are potentially helpful to struggling readers. However, text processing or multimedia learning research has rarely examined learners with low reading abilities (Ozuru et al., 2013) but have focused more often on learners with low prior knowledge (e.g., McNamara et al., 1996; Florax and Ploetzner, 2010). Further, text processing research has distinguished text-based comprehension from inferential comprehension based on their performance measures. Multimedia research has rarely considered this distinction and often assessed comprehension as whole or, in contrast to knowledge transfer, an approach that was derived from research on problem solving. The purpose of this research was to investigate the effects of text cohesion and cross-representational signaling on the comprehension of an instructional text–picture document.

### Learning From Text

Reading with comprehension is not a straightforward process, as the comprehension of even the shortest text may require the generation of inferences. This critical ability is well described in text comprehension research, particularly in the construction–integration model from Kintsch (1998), the original model of which, from Kintsch and van Dijk (1978), was a schema theory. This means that learners have an *a priori* general idea of what they will read about and that the reading will provide them with new information that will feed their schema of the situation. The model then evolved to consider not only instructional texts but narratives as well (Kintsch, 1980), with an emphasis on text structure and its correspondence with the construction of a mental model. In this updated model, a learner integrates the following into a coherent mental model: the elements from the text-based representation, knowledge from long-term memory, and any inferences they make from the text. The ability to generate inferences depends on reasoning processes both to establish the implicit connection between two (or more) pieces of information distributed in the text (bridging inferences) and to build on previous knowledge of the world in order to understand the global situation (elaborative inferences). Therefore, the generation of inferences allows the reader to link elements and build new knowledge. With bridging inferences, learners link provided in neighbored sentences (local bridging inferences) or distributed further apart in the text (global bridging inferences). With elaborative inferences, learners retrieve details from prior knowledge and integrate them in their mental model.

A large body of text comprehension research has demonstrated that learners’ abilities to generate inferences from text were a strong predictor of their success or failure in comprehending the text (Cain and Oakhill, 1999, 2014), independent of individual factors such as word decoding skills, working memory capacity, and domain knowledge. Other research on the influence of the generation of inferences investigated the effects of varying the level of cohesion in a text (McNamara et al., 1996; McNamara, 2001, 2011; Ozuru et al., 2009). McNamara et al. (1996) found that high school students (11–15 years) with low prior knowledge better comprehended a text at high cohesion than at low cohesion and that a reversed effect was observed for high prior knowledge students. Further, this effect was observed for the inferential but not the text-based level of processing. In a study with college students, Ozuru et al. (2009) investigated the interactions between cohesion and prior knowledge, and cohesion and reading abilities. They found that while cohesion in itself did not affect comprehension, it interacted with reading abilities depending on the level of comprehension considered. Regression analyses indicated that the contribution of prior knowledge increased when comprehension required more integration, while an opposite pattern was observed for reading abilities. In other words, low prior knowledge learners particularly benefit from high cohesion for the generation of global inferences, while learners with low reading abilities particularly benefit from high cohesion for the retention of text-based information and the generation of local inferences. Learners with high reading abilities or high prior knowledge are able to generate inferences without support. However, further analyses of variance showed that learners with low reading abilities and low prior knowledge did not benefit from high cohesion, stressing the importance to take both reading abilities and prior knowledge into consideration. Additionally, this line of research (e.g., Ozuru et al., 2009; McNamara, 2011) underlined the importance of distinguishing between the comprehension of elements extracted directly from the text (assessed with text-based questions) and the comprehension of elements requiring the generation of inferences (assessed with local-bridging, global-bridging, and elaborative questions). This body of research found that, overall, facilitating the generation of inferences through manipulations of text cohesion affected only inference questions and particularly so for global-bridging questions.

### Learning From Text and Picture

Current models of multimedia learning (Mayer, 2014; Schnotz, 2014) are based on the dual coding theory (Paivio, 1971; Clark and Paivio, 1991), which predicts—and proved—better memory after a presentation using both a picture and a verbal label of an object than after one using twice the information in one medium. Having information anchored in two representation channels rather than in one results in the construction of a stronger mental model. Both the cognitive theory of multimedia learning from Mayer (2005, 2014) and the latest version of the integrated model of text and picture comprehension (ITPC model) from Schnotz (2014) assert that information from verbal and pictorial representations is first processed through different sensory modalities before being encoded in a coherent model of the situation that relies on both those representations and previous knowledge. According to the cognitive theory of multimedia learning, the multimedia effect, that is, text–picture material, improves learning more than text alone because complementary information provided by the pictorial representation supports the construction of the mental model of the situation, which is required for deep understanding and thus learning (for a more extensive explanation, see for example Schüler et al., 2013). The ITPC model includes a coherence principle, which suggests that “students learn better from words and pictures than from words alone if the words and pictures are semantically related to each other” (Schnotz, 2014, p. 23), especially students with poor reading skills or little prior knowledge. This assertion is based on research showing that learning with multiple representations (particularly written text and pictures) can be beneficial for comprehension, provided that learners can identify the links between representations through cross-references.

Whereas the literature has repeatedly reported that adding pictures to text improves comprehension (e.g., Mayer and Gallini, 1990; Hegarty and Just, 1993; Schnotz and Bannert, 2003; Mason et al., 2013c), there is also evidence that the integration process can be challenging for students (Ainsworth et al., 2002). According to the Design, Functions, Tasks (DeFT) framework (Ainsworth, 1999, 2006; Ainsworth and Van Labeke, 2002), multiple representations can primarily be used to complement one another, constrain the interpretation of each other, or allow learners to construct a deeper understanding of a given topic. Based on the coherence principle from the ITPC model, written text and pictures should be considered as complementary multiple representations, for which “a single representation would be insufficient to carry all the information” (Ainsworth, 1999, p. 137). In addition to the overlap of information across representations, the DeFT framework includes the idea that representations can bear different computational weights (Larkin and Simon, 1987). In short, using computationally unequal representations can be beneficial to learners because they will infer some information more easily from one type of representation than from the other. Using multiple representations with written text and visual pictures, Larkin and Simon (1987) considered that a picture can represent linked information spatially closer together than a written text and thus be more facilitative of the inference generation process.

To guide the integration process of learning material employing multiple representations, multimedia research has investigated the effect of the insertion of visual or verbal cues in either verbal or pictorial representations or both (for reviews, see van Gog, 2014 and Richter et al., 2016). Whereas van Gog (2014) distinguished signals according to their implementation—text based, picture based, or used across representations—Richter et al. (2016) classified signals according to their nature—with verbal signals opposed to visual signals. Verbal signals are *deictic* references that correspond to an explicit reference to the pictorial representation in the text or verbal labels inserted in the pictorial representation. Visual signals refer to the use of a single color for a word in the text and its visual counterpart in the pictorial representation or to the use of color or a spotlight in the picture. Therefore, we consider that the taxonomies of Richter et al. and van Gog should be used concurrently to define or design multiple representations. Results from the meta-analysis by Richter et al. showed an overall significant beneficial effect of signaling in text–picture relations that was more profitable to learners with low and medium prior knowledge than those with high prior knowledge, in line with the ITPC model’s predictions (Schnotz, 2014).

Multimedia learning research has investigated not only learner’s ability to construct a coherent mental model but other characteristics as well. Thus, similar to the results of the text research, multimedia learning research pointed out an expertise reversal effect (Sweller et al., 2003; Kalyuga, 2014), which states that learners with low prior knowledge are more likely to benefit from the text’s adjunct visual representation than learners with high prior knowledge are. Notably, this expertise reversal effect was found in studies on signaling (Spanjers et al., 2011; Richter et al., 2017; Richter and Scheiter, 2019). Moreover, the cognitive–affective theory of multimedia learning (Moreno, 2005, 2006) addressed the question of a link between affect and comprehension. Based on the cognitive–affective theory of multimedia learning, recent work on implementing emotional design (Um et al., 2012; Mayer, 2014; Mayer and Estrella, 2014) focused on the influence of motivation, of which interest is a component (Hidi, 2006). As an example, Um et al. (2012) investigated the effect of adding emotionally positive graphic design elements, such as colors and faces, and found that they increased comprehension, self-rated motivation, and satisfaction. Using a similar manipulation, Mayer and Estrella (2014) found an increased comprehension but no effect on motivation and difficulty ratings. Focusing on the type of picture adjunct to the text, Lenzner et al. (2013, Study 3) found that higher interest was reported when the text was presented with an instructional picture than with a decorative picture. The meta-analysis from Schneider et al. (2018) found an overall effect of signaling on motivation and cognitive load. The inclusion of signaling in single or multiple media positively correlated with motivation and cognitive load, indicating more motivation and less cognitive load when learning with signaled material. Désiron (unpublished) investigated learner characteristics predicting multimedia learning when distinguishing text-based from bridging inference questions (local and global). Findings from this study indicated that different reading abilities (vocabulary and verbal reasoning) affect multimedia comprehension depending on the type of question (text based, bridging inference) asked. Learners’ situational interest was investigated as well and was found not to be a predictor of multimedia comprehension, either for text-based or bridging-inference questions.

Following the theory and research on signaling in multimedia learning, this study investigated the effect of cross-representational signaling (CRS) on learning from a multimedia document. We define CRS as signals supporting a high semantic overlap between written text and visual pictures, following Schnotz’s (2014) ITPC model coherence principle. Following the taxonomy from van Gog (2014), these signals are used across representations, and they are verbal (in both the text and picture) or visual (in the picture), according to the taxonomy from Richter et al. (2016). Indeed, the use of verbal signals in the picture successfully improved comprehension in previous research, particularly in an eye-tracking study by Mason et al. (2013a). Adding color to a pictorial representation was recurrently found to benefit learner comprehension (Jamet et al., 2008; Boucheix et al., 2013), as was using color across text and pictorial representations as well (Kalyuga et al., 1999). More recently, Richter and Scheiter (2019) compared the effect of signaling within the verbal representation with that of signaling across verbal and pictorial representations when learning from a digital chemistry textbook. The authors found that young adults (13–17 years old) with low prior knowledge recalled more information when signaling was used across verbal and pictorial representations but that it did not affect learners with high prior knowledge. However, the manipulation failed to influence the outcome regarding learner comprehension. Based on predictions from the ITPC model (Schnotz, 2014) and the results of previous research, CRS should positively support comprehension when learners have low prior knowledge or reading abilities and the text is difficult to comprehend (low cohesion).

Although multimedia learning research has investigated comprehension, it has not often, or not clearly, distinguished between comprehension of the text base and comprehension requiring the generation of inferences. Rather, it often has focused on the distinction between text-based and transfer questions (e.g., Mayer et al., 2004; de Koning et al., 2011; Mason et al., 2013b). Butcher (2006) investigated learning outcomes with different measures, when the learner was reading a text only, a text with a simplified diagram, or a text with a detailed diagram on the heart and circulatory system. In Experiment 1, learning was measured by means of drawings, memory questions (similar to retention), and inferences (elaborative). These elaborative inferences were close to a transfer task, as learners were asked to transfer knowledge acquired from the instructional document to novel situations. No significant differences between groups were observed for inference questions, but learning with diagrams did lead to the generation of significantly more correct inferences in self-explanation. The generation of inference is still rarely investigated in multimedia research with text and pictured. However, we believe that an assessment distinguishing between text-based and inferential comprehension would greatly benefit the field.

### Research Aim and Hypotheses

The aforementioned literature showed that the comprehension performance of learners with low reading abilities or low prior knowledge was improved by increasing text cohesion or by signaling links between verbal and pictorial information through visual cues in the text and the pictures. However, no study has investigated how these two factors would interact. As we aimed to focus on learners with low prior knowledge and/or low reading abilities, bridging inferences were studied, but elaborative inferences were not.

#### Hypothesis 1

Our first prediction was based on results from text processing research (e.g., McNamara et al., 1996; Cain and Oakhill, 1999, 2014; McNamara, 2001; Ozuru et al., 2009) on the effect of cohesion on text comprehension. Thus, we expected that learners with low prior knowledge reading a highly cohesive text would obtain better scores on inference generation questions than learners with a low cohesive text would obtain.

#### Hypothesis 2

According to the ITPC model (Schnotz, 2014), pictures can be used as guides to comprehend a text. Further, research has showed that the multimedia effect is more salient in learners with low prior knowledge, especially when signaling is used (Richter et al., 2017). Thus, our main prediction was that learners who studied the multimedia material with CRS would perform better than learners who studied the multimedia material without signaling. As pictures should support inference generation, differences were particularly expected in answers to questions requiring the generation of inferences.

#### Hypothesis 3

Finally, we expected that text cohesion would interact with signaling. The highest comprehension performance should be found when the multimedia material is written at a low level of cohesion and includes CRS. The positive effect of CRS should be less pronounced with high text cohesion. Indeed, the guidance from CRS probably does not improve the generation of inferences beyond the benefits of high text cohesion. Previous research has showed that the generation of inferences is supported by both verbal references in the text and signaled visual references in the picture, both of which were found to support comprehension individually (e.g., McNamara, 2001; Richter et al., 2017). Therefore, we expected the interaction to be observed only when the generation of inferences was required and not for text-based questions.

## Experiment 1

### Materials and Methods

#### Participants and Design

Six classes of students (*n* = 95) in first year of one Ecole de Culture Générale (prevocational track) took part in the experiment as part of a class activity proposed by their teacher. The number of participants was determined from an *a priori* power calculation using the software G^∗^Power 3.1 (Faul et al., 2007) for a multivariate analysis of variance (effect size *r* = 0.29—derived from Seufert, 2003, α = 0.05, power of 0.80), with a recommended sample size of 89. Students who did not give their informed consent were given a silent reading task by their teacher. Four participants did not complete all tasks in time and were excluded from the data analyses. The data from 91 participants (51 female) with a mean age of 16.8 years (*SD* = 11 months) were analyzed. This study was approved by the university’s ethics committee and by the school research committee.

Participants were randomly assigned to one of four experimental between-subjects conditions resulting from a two-cohesion (low vs. high) by two-signaling (no signal, with CRS) factorial design.

#### Material

The experimental material was a five-page-long multimedia document on river sailing and how to escape the Maytag effect when caught in a rapid. It was presented in landscape format with the text on the left side and a picture on the right side.

##### Cohesion

The text was written from multiple sources and manipulated to obtain a low and a high cohesive versions. Changes were implemented following the recommendations from Ozuru et al. (2009), which impacted both local and causal inference generation:

1. Replacing ambiguous pronouns with nouns;
2. Adding descriptive elaborations that link unfamiliar concepts with familiar concepts;
3. Adding connectives to specify the relationships between sentences or ideas;
4. Replacing or inserting words to increase the conceptual overlap between adjacent sentences;
5. Adding topic headers;
6. Adding thematic sentences that serve to link each paragraph to the rest of the text and overall topic; and
7. Changing sentence structures to incorporate the additions and modifications (p. 232).

The low cohesive version was 500 words long while the high cohesive version was 706 words long. Supplementary Appendix A contains an example of the low and high cohesive versions of the text in which the specific changes are indicated.

##### Signaling

**FIGURE 1:**
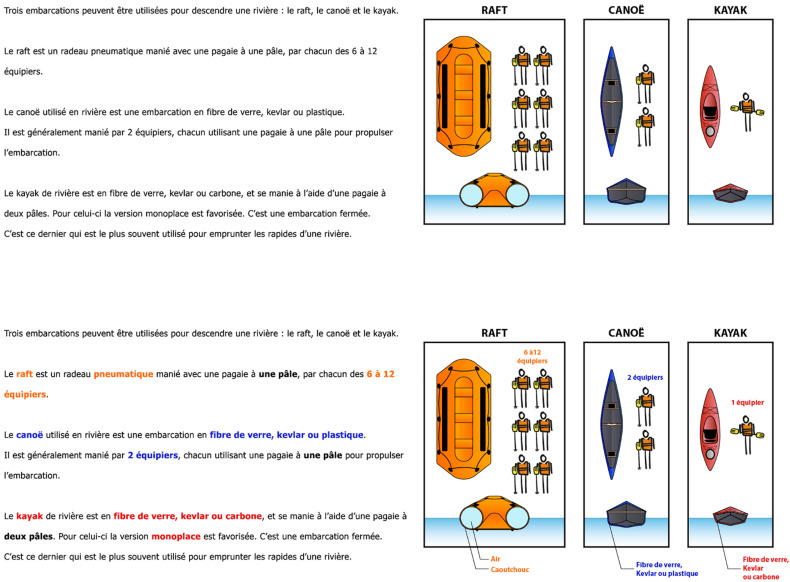
Sample page of the instructional document without signaling **(top)** and with CRS **(bottom)**.

The implementation of signaling in the form of CRS consisted of the insertion of captions and arrows in the picture as well as the use of the same color coding in both the text and the picture. Figure 1 shows an example of a page without signaling and with CRS.

#### Measures

##### Prior knowledge

To control for participants’ knowledge on the topic of river sailing, we used a questionnaire with six statements on a five-item self-rating scale ranging from “do not know” to “know very well” (e.g., *I [*…*] the dangers of river sailing*) or from “cannot explain” to “can explain very well” (e.g., *I [*…*] what a Whitewater is*).

##### Reading abilities

To control for participants’ reading abilities, we used two tests of reading abilities found to be good predictors of multimedia comprehension (Désiron et al., 2018). The vocabulary test was a French version of the Hill Mill assessment, which asks participants to determine 33 synonyms with six options each and an 8 min time limitation (Deltour, 1993), for a maximum possible score of 44 points. The verbal reasoning test was a translation of the test designed by Meteyard et al. (2015) that assesses participants’ ability to generate inferences by means of short texts followed by four open-ended questions, for a maximum possible score of 12 points.

##### Comprehension

In accordance with research on text processing (e.g., Ozuru et al., 2009), comprehension was assessed at three different levels, with short open-ended questions. The findings of Ozuru et al. (2013) indicated that open-ended questions are a more sensitive measure of inference generation than multiple-choice questions. Five text-based questions measured participants’ retention of elements clearly stated in the text (e.g., “Which watercraft[s] use[s] a single-paddle?”) and could be answered with single words. Four local inference questions measured participants’ comprehension of elements that required the generation of bridging inferences from elements no more than a sentence apart (e.g., “Does the Maytag whirlpool form upstream or downstream from the boiling?”). Four global inference questions measured participants’ comprehension of elements that required the generation of bridging inferences from elements dispersed in the text (e.g., “According to the document, why is it only after the boiling that one should resurface?”). The local and the global inference questions needed to be answered with one or two sentences. Therefore, the answers that were expected for comprehension questions ranged from one word to two sentences, depending on the level of comprehension. Utilizing an analysis grid taking into consideration idea units from the text and pictures, each question could score 1 point, with the value of the idea unit ranging from 0.50 to 1, depending on the number of ideas expected. Thus, the score per level of comprehension thus ranged between 0 and 4 (inference questions) or 0 and 5 (text-based questions). The answers were evaluated by the first author, and a second rating was done by the second author on a random subset of 20% of the comprehension questions (*n* = 20). Interrater reliability was determined by intraclass correlation coefficients (ICCs), which were ICC (3, 1) = 0.93 for the comprehension questions. The raters jointly settled their few differences in the two ratings.

#### Procedure

This experiment was conducted in school, during 45 min classes, with up to 10 participants using 9.7-in tablets. The participants first completed the self-assessed knowledge questionnaire, before reading the experimental material in one of the four experimental conditions (low cohesion no signal, *n* = 23; low cohesion with CRS, *n* = 23; high cohesion no signal, *n* = 22; high cohesion with CRS, *n* = 23) without a time limit. The participants were then prompted to answer the comprehension questions, presented following the order in which the elements requiring an answer occurred in the text. Finally, the participants completed the reading ability tests, presented in a random order. At the end of the allocated time, the participants were debriefed with regards to the research hypotheses corresponding to the different experimental conditions.

### Results

We used a 2 × 2 factorial design with cohesion (low vs. high) and signaling (none, CRS) as the between-subjects factors and comprehension questions (text based, local inferences, global inferences) as the dependent variable.

#### Learner Characteristics

The sample as a whole had little prior knowledge (*M* = 5.53 out of 24, *SD* = 3.89). Overall, the participants scored just above half of the maximum possible points on the vocabulary test (*M* = 23.84, *SD* = 4.25), which was below the expected score for their age range (Deltour, 1993). Regarding the test of verbal reasoning, the participants scored about half of the possible points (*M* = 6.70, *SD* = 2.18). Therefore, this sample corresponded to the conditions deemed more likely to benefit from multimedia documents, according to the ITPC model (Schnotz, 2014).

To control for an effect of participants’ characteristics, we ran a correlation analysis of the three levels of comprehension. Prior knowledge did not correlate with any level of comprehension, vocabulary correlated with all levels (*p* < 0.001), and verbal reasoning correlated with text-based and local inferences comprehension questions (*p* = 0.002 and *p* = 0.032, respectively). The statistical analyses also indicated that covariates were not significantly different across groups (prior knowledge, *p* = 0.155; vocabulary, *p* = 0.806; and verbal reasoning, *p* = 0.135).

#### Effects of Cohesion and Signaling on Comprehension

A multiple factor analysis of covariance (MANCOVA) was performed on comprehension scores with cohesion level (low, high) and signaling (no signal, with CRS) as the between-subjects independent variables and comprehension questions (text based, local inferences, global inferences) as the dependent variable. Following correlation analysis (see the previous section for details), reading ability tests for vocabulary and verbal reasoning were included as covariates. As shown in Table 1, there was no significant advantage of high cohesion, *V* = 0.084, *F*(3, 83) = 2.53, *p* = 0.063, η^2^_*p*_ = 0.084, but there was a significant trend in local inferences questions for those who learned with a highly cohesive text (*M* = 1.36, *SD* = 0.91) compared to those who learned with low cohesive text (*M* = 1.02, *SD* = 0.75), *F*(1, 85) = 6.29, *p* = 0.014, η*^2^_*p*_* = 0.069.

**TABLE 1:** Estimated marginal means and standard errors for the outcome measures in Experiment 1 (*n* = 47).

	No signal		With CRS	
	EMM	SE	EMM	SE
**Low cohesion**				
Text-based questions (max score 5)	1.96	0.18	2.08	0.18
Local inferences questions (max score 4)	0.89	0.16	1.04	0.16
Global inferences questions (max score 4)	0.66	0.15	1.06	0.15
**High cohesion**				
Text-based questions (max score 5)	2.22	0.18	2.15	0.18
Local inferences questions (max score 4)	1.15	0.16	1.16	0.16
Global inferences questions (max score 4)	0.65	0.15	0.94	0.14

*Maximum score indicated in parentheses.*

Consistent with Hypothesis 2, there was a significant effect of signaling, *V* = 0.092, *F*(3, 83) = 2.79, *p* = 0.046, η*^2^_*p*_* = 0.092, and learners with CRS scored higher than learners without signaling on local (*M* = 1.04, *SD* = 0.74 no signal; *M* = 1.33, *SD* = 0.92 with CRS) and global (*M* = 0.67, *SD* = 0.56 no signal; *M* = 1.01, *SD* = 0.88 with CRS) inferences questions. Separate two-factorial analyses of covariance on the outcome variables revealed a significant effect for global inferences questions, *F*(1, 85) = 2.84, *p* = 0.017, η*^2^_*p*_* = 0.065. The difference for local inferences questions was only a marginally significant trend, *F*(1, 85) = 1.92, *p* = 0.072, η*^2^_*p*_* = 0.038. As expected, there was no significant effect of signaling for text based questions (*p* = 0.898).

There was no significant multivariate effect of the interaction between cohesion and signaling, *V* = 0.019, *F*(3, 83) = 0.55, *p* = 0.649, η*^2^_*p*_* = 0.019. The covariate, vocabulary, was significantly related to comprehension questions for text based, *F*(1, 78) = 29.79, *p* < 0.001, local inferences, *F*(1, 78) = 15.17, *p* < 0.001, and global inferences, *F*(1, 78) = 17.08, *p* < 0.001. The covariate, verbal reasoning, was significantly related to comprehension questions for text based, *F*(1, 78) = 8.11, *p* = 0.006, but not to local inferences, *p* = 0.126, and global inferences, *p* = 0.479.

### Discussion of Experiment 1

This first experiment tested three hypotheses on the effect of text cohesion and CRS on multimedia comprehension. In line with text processing research, three levels of comprehension (text based, local inference, and global inference) were assessed. In addition, some learners’ characteristics that were found to affect text comprehension were measured. In this regard, the results concurred with previous findings on the role of language skills (vocabulary and to a lesser extent verbal reasoning) in the comprehension score for multimedia learning (Désiron, unpublished). There was no effect of prior knowledge on the comprehension score, probably because the knowledge level overall was very low. Regarding the effect of the two independent variables, the results hardly supported Hypothesis 1, as there was no effect of cohesion on comprehension scores overall. Still, an effect of cohesion for the generation of local inferences was observed. The findings partially supported Hypothesis 2 because a significant positive effect of CRS was found for global inferences and a positive marginal trend for local inferences. No difference was found for text-based scores, as expected. Finally, Hypothesis 3 was not supported, with no significant interaction between the two independent variables.

The beneficial role of CRS is in line with a previous research (Richter et al., 2016) that found a positive effect of signaling on multimedia comprehension. This confirms the assumption of the ITPC model (Schnotz, 2014), which posits that students with low prior knowledge and reading abilities, as were those in the sample of this study, benefit from support to connect the corresponding verbal and pictorial information. However, the underlying mechanisms and processes explaining this effect are still speculative and need further investigation, which is presented in Experiment 2. Regarding cohesion, previous researches have provided mixed results (Ozuru et al., 2009; McNamara et al., 2011) that was explained by some variability across studies of the sample under consideration (in particular their reading abilities and prior knowledge) and the ways to concretely implement cohesion in the material. Indeed, increasing cohesion also affects text length, which may factor as another difficulty for learners with very low reading abilities. For these reasons, the cohesion factor was not varied in the next experiment. Only the low cohesion version was kept because, according to the ITPC model, the effect of CRS on comprehension is more likely to appear when the text is difficult and most available texts were written at this level (Graesser et al., 2004).

## Experiment 2

In the second experiment, offline outcome measures of multimedia comprehension were combined with online measures, using eye tracking, and measure of learners’ subjective evaluations (motivation and cognitive effort), as it is often practiced in multimedia learning research (Paas et al., 1994; Mayer and Estrella, 2014; Huang and Mayer, 2016). In addition, comprehension was assessed with a drawing task, which is, to our knowledge, rarely used to assess inference generation in multimedia research. For example, Butcher (2006) asked learners to draw about the heart and circulatory system as a pre- and posttest assessment of their mental models.

Previous multimedia research (see Richter et al., 2016 for a review) and the results from our first experiment showed that signaling successfully supported this integration process for struggling readers. However, the integration process of verbal and pictorial representation is not only reflected by offline measures of inferential comprehension but also by online measures such as gaze data as well (e.g., Mason et al., 2013a). The seminal eye-tracking study from Yarbus (1967), who compared picture-free observation and observation with instructions, demonstrated that eye movements reflected attention to visual material and thus top–down or bottom–up processing. The aim of inserting signaling devices in multimedia documents is to guide learners’ attention by prompting top–down processing. Signals are assumed to support learners’ selection of information and particularly so when they have little prior knowledge of the content (Ozcelik et al., 2010; van Gog, 2014). For example, Mason et al. (2013a) compared the use of labels in a picture adjunct to a text on atmospheric pressure in a sample of sixth graders and found that the presence of signals effectively supported text–picture integration. Further, eye-tracking data revealed longer fixations on signaled elements (labeled in the picture) of both the text and picture during text rereading or picture reinspection (second pass) when labels were included. Scheiter and Eitel (2015) studied the effect of colored labels in both the text and picture on learning about the heart and circulatory system. Their results indicated that learners directed more frequently their attention toward the information when it was signaled than when it was not. Previous research also has showed that multimedia manipulations affect not only learning outcomes but the learners as well, as hypothesized by the cognitive–affective theory of multimedia learning (Moreno, 2005, 2006). The effects of the implementation of multimedia principles were found to impact motivation (Mayer and Estrella, 2014; Dousay, 2016; Schneider et al., 2018) and cognitive effort (Huang and Mayer, 2016). The meta-analysis from Schneider et al. (2018), in particular, showed that signaling positively affected motivation, although the effect was small. Based on Experiment 1 and the literature, we expected to confirm Hypothesis 2, which posits that CRS is beneficial for comprehension at the inference level. Moreover, eye-tracking measures were used to determine whether the beneficial effect of CRS was linked to the fostering of attention toward the pictorial information, and the text–picture integration following the procedure used in Mason et al. (2013a) and Scheiter and Eitel (2015). This second experiment also explored the effect of CRS on motivation and cognitive load as outcome variables. Prior knowledge of the topic and reading abilities were still used as control measures.

### Materials and Methods

#### Participants

Forty-seven bachelor’s degree students in social sciences (35 female) with a mean age of 22.3 years (*SD* = 3 years) took part in the experiment. The participants were recruited through online selection based on their prior knowledge. Only applicants with a prior-knowledge score under 11 out of 24 corresponded to the target population and participated with a financial compensation (20 CHF). This study was approved by the University of Geneva ethics committee.

#### Apparatus and Material

The experimental material used was the same as in Experiment 1. However, based on the results of the first experiment, only the low cohesion version of the text was used.

##### Eye-tracking equipment

Eye movements were recorded using a Tobii TX300 eye tracker (Tobii Technology, Stockholm, Sweden). The eye tracker was connected to a 23-in monitor with a maximum resolution of 1,920 × 1,080 pixels. Pupil location and pupil size were sampled at a rate of 300 Hz, and data were recorded with Tobii-Studio software.

#### Measures

The measures of prior knowledge, reading abilities, and comprehension were identical to those of Experiment 1. Please refer to section “Material” in “Experiment 1” for detailed information.

In this second experiment, answers to the comprehension questions were evaluated by the first author, and a second rater randomly evaluated a subset of 20% of the comprehension questions (*n* = 17). Interrater reliability was determined by ICCs, which were ICC (3, 1) = 0.87 for the comprehension questions. The few differences in the two ratings were jointly settled by the raters (α = 0.60). Aside from the open-ended questions, comprehension of page 3 of the document was assessed with a drawing task. The participants were provided with a background picture of a riverbed, which was the same as in the document, and were asked to draw and name the three currents involved in the formation of a white water. Each element was graded with respect to its position (e.g., located before the change of slope) and representation (e.g., drawn as bubbles), with 1 point per element and a total of 3 points for the task.

##### Attitude

In addition to the measures from Experiment 1, the participants completed the student attitude survey adapted and translated from Huang (2017), containing four statements on motivation (e.g., “I liked studying about the dangers of the river rapids.”), three on perceived difficulty (e.g., “Studying the dangers of the river rapids, by myself, was a difficult way to learn.”), and three on perceived effort (e.g., “I did my best to learn about the dangers of the river rapids.”), with a seven-item Likert scale ranging from “do not agree at all” to “fully agree.”

##### Eye tracking

**FIGURE 2:**
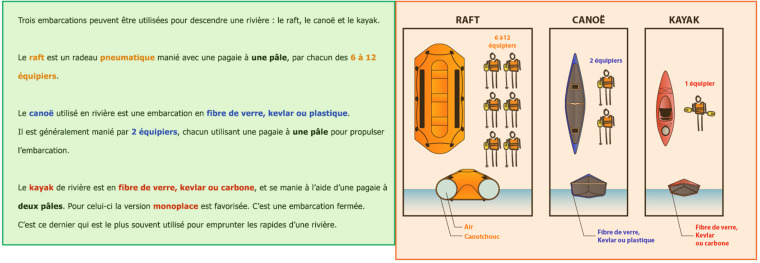
Sample page of the instructional document with areas of interest (AOI).

Similar to the study by Dessus et al. (2016), ratios were computed for analyses rather than using raw values to compare variances in the observed values because there was no time limitation to complete the reading activity. Thus, for eye-tracking data analyses, each page of the document was divided in two areas of interest (AOIs), the text and picture areas (see Figure 2 for an example), and ratio values were computed for the number of fixations, duration of fixations, and gaze length on pictures (i.e., uninterrupted sequences of fixations within an AOI), relative to the summed data for both AOIs. In other words, participant’s time-related data were the ratio of time spent on the picture AOI relative to the overall time spent on a page. The number of transitions from text to picture (Mason et al., 2015) was computed with the R statistics program, based on eye-tracking logs indicating whether or not there was a fixation in an AOI. Therefore, for each participant, a transition consisted in searching for subsequent rows in which the first row had the text AOI activated and the following row had the picture AOI activated. Eye movements outside the two AOIs were excluded from data analyses.

#### Procedure

During the recruitment phase, the participants who corresponded to the target population were asked to complete the reading ability test for vocabulary. A median score was then computed before participants’ laboratory session, taking into account all registered participants up to the time of the session. To ensure variability of reading abilities between signaling conditions (none vs. CRS), participants were alternatively assigned to one of the two conditions based on the latest median split result available. The main part of this experiment was conducted in our laboratory with one participant at a time. The participants first read the experimental material, without time limit. They were then asked to complete the attitude survey before answering to the comprehension questions. Finally, the participants completed the verbal reasoning test for reading abilities. At the end of their session, the participants were debriefed regarding the research hypotheses and differences between the experimental conditions.

### Results

#### Learners’ Characteristics

As participants were selected based on their low prior knowledge, the sample as a whole scored rather low on this test (*M* = 3.87 out of 24, *SD* = 2.86). Overall, the participants’ mean score to the vocabulary test was 33.06 (*SD* = 3.73), which was between the expected 25th and 50th percentile for their age range (Deltour, 1993). Regarding the test for verbal reasoning, the participants scored more than half of the possible points (*M* = 7.73, *SD* = 1.52). Therefore, this sample matched the little prior knowledge condition but not the low reading ability condition, under which the multimedia effect is more likely to occur, according to the ITPC model (Schnotz, 2014).

To control for an effect of participants’ characteristics, we ran a correlation analysis with measures of comprehension (the three types of questions and the drawing task). Prior knowledge and vocabulary did not correlate with measures of comprehension, whereas verbal reasoning correlated with local inferences questions (*p* = 0.045) but not with the other measures of comprehension (text-based and global inferences questions, and the drawing task). The statistical analyses also indicated that the covariates were not significantly different across groups (prior knowledge, *p* = 0.622; vocabulary, *p* = 0.688; and verbal reasoning, *p* = 0.451).

#### Effects of Signaling on Comprehension

A MANCOVA was performed on comprehension scores with signaling (none, CRS) as the between-subjects independent variable and comprehension scores (text based, local inferences, global inferences) as the within-subjects dependent variables. Based on the correlation analysis reported above, the reading ability score for verbal reasoning was included as a covariate. Consistent with Hypothesis 1, the participants in the CRS condition had higher scores to the comprehension questions than the participants in the no signaling condition (Table 2); however, the differences did not reach statistical significance, *V* = 0.033, *F*(3, 42) = 0.47, *p* = 0.703, failing to replicate the results of Experiment 1.

**TABLE 2:** Estimated marginal means and standard errors for the outcome measures in Experiment 2 (*n* = 47).

	No signal		With CRS	
	EMM	SE	EMM	SE
**Comprehension**				
Text-based questions (max score 5)	3.02	0.19	2.94	0.18
Local inferences questions (max score 4)	1.48	0.18	1.64	0.18
Global inferences questions (max score 4)	1.45	0.20	1.70	0.19
Drawing task (max score 3)	1.68	0.14	1.92	0.13
**Attitude**				
Motivation (max score 28)	24.26	0.81	23.50	0.80
Perceived difficulty (max score 21)	7.13	0.62	7.75	0.61
Perceived effort (max score 21)	14.35	0.46	14.25	0.45

*Maximum score indicated in parentheses.*

For the drawing task, we performed an analysis of variance (ANOVA) with signaling (none, CRS) as the between-subjects independent variable and the drawing task as the dependent variable. There was no significant effect of signaling, *F*(1, 45) = 1.46, *p* = 0.223.

#### Effects of Signaling on Text–Picture Integration

Due to poor eye calibration, six participants were excluded from the eye-tracking data analyses. The data of 41 participants (32 female) with a mean age of 22.2 years (*SD* = 3 years) were analyzed. Descriptive values for the eye movements are shown in Table 3.

**TABLE 3:** Mean and standard deviations for eye-movement data in Experiment 2 (*n* = 42).

	No signal		With CRS	
	*M*	*SD*	*M*	*SD*
Fixations count on picture (ratio)	0.20	0.06	0.26	0.07
Fixations duration on picture (ratio)	0.19	0.07	0.26	0.08
Gazes length on picture (ratio)	0.20	0.07	0.26	0.09
Transitions from text to picture	22.37	9.78	23.41	11.48

We first analyzed the ratio of the number of fixations on the picture as a function of the signaling (none vs. CRS). An ANOVA revealed a significant effect of signaling, *F*(1, 39) = 7.83, *p* = 0.008, η*^2^_*p*_* = 0.167, with more fixations on the picture with CRS than without signaling. Second, we analyzed the ratio of the sum of the duration of fixations on the picture as a function of the signaling (none vs. CRS). An ANOVA revealed a significant effect of signaling, *F*(1, 39) = 7.80, *p* = 0.008, η*^2^_*p*_* = 0.167, with longer fixations on the picture with CRS than without signaling. Third, we analyzed the sum of gaze lengths on the picture as a function of the signaling condition (none vs. CRS). An ANOVA revealed a significant effect of signaling, *F*(1, 39) = 8.19, *p* = 0.007, η*^2^_*p*_* = 0.173, with longer gazes on the picture with CRS than without signaling. Finally, we analyzed the number of transitions from text to picture as a function of the signaling condition (none vs. CRS). An ANOVA did not yield any significant effect of signaling for the number of transitions, *F* (1, 39) = 0.36, *p* = 0.552. Overall, these results are in line with previous findings in multimedia research (e.g., Scheiter and Eitel, 2015) regarding picture processing when using signaling, with more attention paid to the picture in the signaling condition than in the no signal condition.

#### Effect of Signaling on Motivation and Cognitive Load

There was no difference between the CRS or no signal conditions for either motivation, *t*(45) = 0.67, *p* = 0.507, perceived difficulty, *t*(45) = -0.71, *p* = 0.477, or perceived effort, [45) = 0.15, *p* = 0.879. Overall, the learners reported high motivation (*M* = 23.90, *SD* = 3.87), low perceived difficulty (*M* = 7.45, *SD* = 2.95), and medium perceived effort (*M* = 14.30, *SD* = 2.17).

### Discussion of Experiment 2

The aim of the second experiment was to investigate the underlying mechanisms explaining the beneficial effect of CRS on comprehension as observed in the literature and partially supported in Experiment 1. In particular, the signaled material was expected to foster integrative processes during the study of the material, as observed through eye-tracking measures. Contrary to Experiment 1, there was no effect of CRS on any of the comprehension measures, whether assessed verbally or through drawing. This discrepancy between experiments could be explained by the fact that the participants of Experiment 2 had higher reading abilities than those in Experiment 1. According to the ITPC model from Schnotz (2014), the beneficial effect of supporting text–picture integration is more likely to occur when learners have low reading abilities or low prior knowledge because learners with good reading abilities or high prior knowledge can generate inferences across media without help (Mayer and Gallini, 1990). Nonetheless, the eye-tracking data revealed a significant effect of signaling on visual exploration, with a higher ratio in number, length, and total time of fixations on the picture in the signaled condition. It should be pointed out that the effect size was low, and thus, a larger sample size may be targeted in future studies. No effect of CRS was observed for the number of transitions between text and picture information. Overall, the results concur with the hypothesis that CRS has an impact on visual exploration with more attention being paid to the picture than in a condition without signaling.

## Discussion

This research addresses three hypotheses: (a) struggling readers’ comprehension is higher when learning from a multimedia document with high text cohesion than one with low text cohesion, (b) their comprehension is higher when the multimedia document includes signaling than when it does not, and (c) the effects of cohesion and signaling interact, with higher comprehension with CRS especially when text cohesion is low and to a lesser extent when cohesion is high. Theoretical and empirical research on text documents have found that overall learners with low prior knowledge benefit from high text cohesion (e.g., Ozuru et al., 2009; Cain and Oakhill, 2014), while the positive effect for struggling reader is still scarcely investigated and less clear cut (Ozuru et al., 2009). Therefore, one of our goals was to determine whether a similar effect occurred when one is learning from an instructional document including text and pictures. However, following design recommendations from the ITPC model (Schnotz, 2014), when learners have low prior knowledge or reading abilities, text–picture integration ought to be supported to observe a multimedia effect. Thus, we examined the effect of learning with CRS or without signaling. Moreover, we expected that text cohesion and signaling would interact, with learners benefiting more from signaling when text cohesion is low than when it is high. Learning outcomes were assessed using measures developed in the text research. In other words, although multimedia research mostly considers retention and transfer, as in research focusing on problem solving, we assessed three levels of comprehension as is common in text processing research. Therefore, the effects of our dependent variables can be assessed separately for both text-based comprehension (which is close to retention) and inferential comprehension (local and global). Additionally, we explored how signaling affects online processing of the instructional document (Experiment 2).

Regarding the first hypothesis, the findings of Experiment 1 did not show any significant effect of text cohesion overall, although a trend could be observed and a significant positive effect of high cohesion was found for local inferential level of comprehension. Although our sample overall had low prior knowledge and reading abilities, we were unable to replicate the results of previous text research manipulating cohesion.

The lack of effect of cohesion could be explained by the fact that the participants in this study had rather low levels of reading abilities (based on the normed vocabulary scale) and thus were not representative of a wide range of reading abilities mastery. Previous studies reporting an effect of cohesion always found it in interaction with learners’ prior knowledge (e.g., McNamara et al., 1996; McNamara, 2011) or reading abilities (Ozuru et al., 2009). Moreover, Ozuru et al. (2009) found no positive effect of cohesion for college students with both low prior knowledge and low reading abilities. They concluded that prior knowledge more than reading abilities determine learners’ ability to generate inferences. In this study, such interaction could not be studied, and only a trend of a beneficial effect of cohesion on local inference generation was observed in learners with low reading abilities. Therefore, it may be that the effect of cohesion on text and multimedia comprehension should not focus merely on learners with low reading abilities but should investigate how well reading abilities need to be mastered to generate local and global inferences. Or, as hinted by Ozuru and colleagues, although prior knowledge is not required to generate bridging inferences, it may still be a stronger predictor of inferential comprehension than reading abilities. Future research could investigate whether a positive effect of high text cohesion is more likely to occur when learners have reached a certain mastery of reading abilities. Further, multimedia research has rarely used different levels of comprehension, and as stressed by Désiron (unpublished), more research is needed to determine which abilities are in play for each level of comprehension.

Another avenue for explaining the limited effect of cohesion resides in the presence of pictures. Indeed, similar to high text cohesion, pictures overlapping with text content are meant to support inference generation by providing the information in a complementary format (Schnotz, 2014). Therefore, a possible interpretation of the absence effect of cohesion for global inferences is that the pictures provided sufficient support to trigger the generation of such inferences (Holmes, 1987). Although numerous studies from the 1990s and up until now have showed a positive effect of multimedia over text only when assessing retention and transfer (e.g., Mayer and Gallini, 1990; Mason et al., 2013a), more studies with a focus on the different levels of comprehension are needed to test this assumption. Undeniably, the distinction between retention and transfer is tailored to measure learners’ abilities to solve problems. However, retention and transfer may not be fine grained enough measures of comprehension compared to the different levels distinguished by text processing research. Nonetheless, descriptive date from the present study show that, overall, participants got low performance to inferential comprehension questions, which probably result from the rather low reading abilities of the participants in the sample. Therefore, the hypothesis that the presence of picture was more effective to support inference generation than cohesion should be investigated in further research, with a wider range of reading abilities within the sample and different levels of inference generation.

Regarding the second hypothesis, the effect of CRS on comprehension yielded mixed results. In Experiment 1, there was a positive effect of signaling on comprehension overall. Detailed analyses showed that the effect was significant for comprehension with global inferences, but only a marginally significant trend was observed for local inferences. In addition, no effect of signaling was observed at the text-based level, as was expected. In Experiment 2, there was no statistically significant difference between the signaled and unsignaled version for any comprehension measure.

The results from previous research suggested that prior knowledge affects the signaling effect, with a stronger effect in learners with low prior knowledge (Richter et al., 2016). Thus, we designed experimental material for which learners would have little prior knowledge and controlled for this effect. The results from the first experiment therefore are in line with previous research on the signaling effect and also highlight that the effect of signaling mostly influences the generation of global inferences. Regardless, we could not confirm this effect of CRS in our second experiment. In line with aptitude–treatment interaction research (Snow, 1991), an argument can be made that the level of reading abilities is yet another predictor of the signaling effect. Indeed, whereas samples from Experiments 1 and 2 had similar prior knowledge of the topic of Rapids, they greatly differed in their reading ability. In the first experiment, the participants had a low level of reading abilities, and signaling was beneficial to comprehension (local inference generation). In Experiment 2, the participants had a higher level of reading abilities and scored much higher on comprehension assessments compared to the first experiment. In other words, it could be that signaling, in these studies, was superfluous for learners with high reading abilities but helped learners with low reading abilities to generate local inferences, although it was insufficient to support global inference generation. This research does not allow the empirical assessment of this hypothesis, which could be investigated in future research with a larger range of reading abilities in the sample.

Contrary to our third hypothesis, there was no interaction between our independent variables, which is not surprising given the limited effect of cohesion discussed above. Therefore this research cannot make any statement regarding this third hypothesis.

In addition to the three hypotheses on the effects of cohesion and signaling, the second experiment investigated whether the guidance provided by signaling was observable in learners’ visual exploration of the text–picture material. In line with previous research (e.g., Scheiter and Eitel, 2015), the eye-movement data revealed that, when the instructional documents included CRS, more attention was allocated to the picture than when no signaling was used. Even though the learners with CRS did not demonstrate better comprehension than those without signaling, they seem to have processed the picture in greater depth. As research has showed that learners often overlook pictures when reading, our results confirm that CRS is an effective design tool to ensure that learners allocate substantial attention to pictorial information (Hannus and Hyönä, 1999; Schnotz et al., 2014b). However, there was no effect of signaling regarding text–picture transitions, which does not replicate results from previous research showing higher text–picture integration processing behavior (Schnotz et al., 2014a). In line with Scheiter and Eitel (2015), we assume that learners may have relied on their memory of the inspected elements rather than on repeated transitions from one representation to the other because, in the material, the text and picture elements to be integrated were not numerous. Again, we believe that future research could investigate the effect of CRS with a wider range of reading abilities to determine whether its mastery interacts with the signaling effect. A qualitative analysis of exploration behavior, such as the distinction of precise elements (key concepts and their pictorial representation) and when they are attended to would also be very informative. Another avenue for the assessment of text–picture processing and the interpretation of eye-movement behaviors could be the use of think-aloud protocol (e.g., Schellings et al., 2006).

Overall, this research provides insights on the use of cohesion and signaling in non-procedural instructional material, when learning is assessed by considering different levels of comprehension. Based on our results, we can recommend that instructional material be designed with CRS. Ideally, when text–picture documents are presented in a digital format (either on computers or tablets), we recommend that signaling be implemented as an on-demand feature. The idea is that signaling could be activated when learners have low reading abilities or could be used as a tool to gradually learn inference generation. The results from this research provide leads on the design of such text–picture documents all the while pointing out limitations and raising new questions. A limitation to our research is that, although the samples of both studies had similar prior knowledge, they greatly differed in age ranges and reading abilities. The differences between samples most likely influenced our results. Hence, more research is needed to determine more precisely how reader characteristics (prior knowledge, reading abilities) predict the effectiveness of signaling in text–picture documents. Finally, it is likely that, although our results extend to declarative and conceptual topics, they may differ when procedural topics are considered.

## Data Availability Statement

The raw data supporting the conclusions of this article will be made available by the authors, without undue reservation.

## Ethics Statement

The studies involving human participants were reviewed and approved by the Commission d’éthique de la Facculté de Psychologie et Sciences de l’Education de l’Université de Genève. Written informed consent from the participants’ legal guardian/next of kin was not required to participate in this study in accordance with the national legislation and the institutional requirements.

## Author Contributions

JD: conceptualization, methodology, experimental material, formal analysis, investigation, resources, and writing—original draft and visualizations. MB: conceptualization, methodology, experimental material, and writing—review and editing. EV: conceptualization and writing—review and editing. All authors contributed to the article and approved the submitted version.

## Conflict of Interest

The authors declare that the research was conducted in the absence of any commercial or financial relationships that could be construed as a potential conflict of interest.
