# Supplementary material for: Cross-Representational Signaling and Cohesion Support Inferential Comprehension of Text–Picture Documents
Source: Front Psychol. 2021 Jan 18;11:592509. doi: 10.3389/fpsyg.2020.592509 (PMC7847939; doi:10.3389/fpsyg.2020.592509)
Supplement: Supplementary file 1 [file Image_1.PDF]

Supplementary Material

1 Supplementary Data

1.1 Appendix A. Example texts

1.1.1 *A1. Excerpt of the page 1 (low cohesion)*

Trois embarcations peuvent être utilisées pour descendre une rivière : le kayak, le canoë et le raft.

Le raft est un radeau pneumatique manié avec une pagaie à une pâle, par chacun des 6 à 12 équipiers.

Le canoë utilisé en rivière est une embarcation en fibre de verre, kevlar ou plastique. Il est généralement manié par 2 équipiers, chacun utilisant une pagaie à une pâle pour propulser l'embarcation.

Le kayak de rivière est en fibre de verre, kevlar ou carbone, et se manie à l'aide d'une pagaie à deux pâles. Pour celui-ci la version monoplace est favorisée. C'est une embarcation fermée.

C'est ce dernier qui est le plus souvent utilisé pour emprunter les rapides d'une rivière.

1.1.2 *A2. Excerpt of the page 1 (high cohesion)*

La descente de rivière

Trois embarcations peuvent être utilisées pour descendre une rivière : le kayak, le canoë et le raft.

Le raft est un radeau pneumatique**, embarcation en caoutchouc dont les rebords sont emplis d'air,** manié avec une pagaie à une pâle, par chacun des 6 à 12 équipiers.

Le canoë utilisé en rivière est une embarcation en fibre de verre, kevlar ou plastique. *Le canoë* est généralement manié par 2 équipiers, chacun utilisant une pagaie à une pâle pour propulser l'embarcation.

Le kayak de rivière est en fibre de verre, kevlar ou carbone, et se manie à l'aide d'une pagaie à deux pâles. Pour *le kayak de rivière* la version monoplace est favorisée. Contrairement au raft et au canoë *le kayak de rivière* est une embarcation fermée, le kayakiste a les jambes à l'intérieur de l'embarcation.

C'est *le kayak de rivière* qui est l'embarcation la plus souvent utilisée pour emprunter les rapides d'une rivière.

Note: Underlined font represents sections added to increase local cohesion. Font in italics represents changes in sentence structure made to increase local cohesion. Bold font represents sections added to increase global cohesion.
